# Liver safety of tolvaptan in patients with autosomal dominant polycystic kidney disease: interim data from a post-authorization safety study

**DOI:** 10.1093/ckj/sfae324

**Published:** 2024-10-30

**Authors:** Thomas Jaeger, Emanuel Lohrmann, Adachukwu Ezenekwe, Kene Enekebe, Retesh Kumar, Sasikiran Nunna, Ancilla W Fernandes, Linda McCormick, Vinu George

**Affiliations:** Otsuka Pharmaceutical GmbH, Frankfurt, Germany; Otsuka Pharmaceutical GmbH, Frankfurt, Germany; Otsuka Pharmaceutical Development and Commercialization Inc., Princeton, NJ, USA; Otsuka Pharmaceutical Development and Commercialization Inc., Princeton, NJ, USA; Otsuka Pharmaceutical Europe Ltd., Wexham, UK; Otsuka Pharmaceutical Development and Commercialization Inc., Princeton, NJ, USA; Otsuka Pharmaceutical Development and Commercialization Inc., Princeton, NJ, USA; Otsuka Pharmaceutical Development and Commercialization Inc., Princeton, NJ, USA; Otsuka Pharmaceutical Development and Commercialization Inc., Princeton, NJ, USA

**Keywords:** hepatotoxicity, liver, pharmacovigilance, safety, tolvaptan

## Abstract

**Background:**

After the risk of drug-induced liver injury was detected during tolvaptan clinical development for the treatment of autosomal dominant polycystic kidney disease (ADPKD), a post-marketing pharmacovigilance study was required for European Union regulatory approval.

**Methods:**

This is an interim analysis from a prospective, observational study enrolling patients prescribed tolvaptan for ADPKD in routine clinical practice. Data were obtained through physician records collected during regular care. Per the prescribing label, liver transaminases were to be monitored monthly for the first 18 months of treatment and once every 3 months thereafter. Patients and physicians were required to report adverse events suggestive of serious and potentially fatal liver injury. Data collection was from October 2016 to April 2022.

**Results:**

Of 2074 patients (median follow-up 528 days), alanine aminotransferase (ALT) or aspartate aminotransferase (AST) levels ≥3 times the upper limit of normal (ULN) were reported in 75 (3.6%) patients. At data cut-off, the enzyme elevations were confirmed for 65 patients. Among the 65 confirmed patients, in addition to transaminase elevations, there were 69 adverse events suggestive of liver injury. Tolvaptan was interrupted or withdrawn in 59/65 (90.8%) participants with confirmed ALT or AST ≥3 times the ULN, with most transaminase elevations and adverse events resolved or resolving at data cut-off. No liver enzyme elevations met laboratory criteria for Hy's law cases.

**Conclusions:**

Transaminase elevations occurred post-marketing in a similar proportion of patients as reported in clinical trials (4.4–5.6%). Regular monitoring per label facilitates prompt detection of liver adverse events and intervention to mitigate the risk of severe injury.

## Graphical Abstract

**Graphical Abstract:**
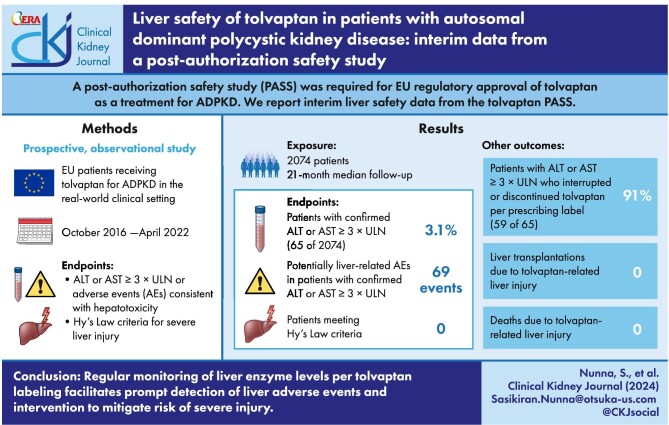

KEY LEARNING POINTS **What was known:**

- During clinical trials of tolvaptan for the treatment of autosomal dominant polycystic kidney disease (ADPKD), transaminase elevations >3 times the upper limit of normal (ULN) occurred in 4.4–5.6% of participants in tolvaptan study arms.
- To manage the risk of liver injury, European Union (EU) tolvaptan labelling requires regular liver chemistry monitoring.
- Clinical trial and interim post-marketing data support regular monitoring to mitigate the risk of severe liver injury, but more real-world data are needed to fully characterize the liver safety profile of tolvaptan.

**This study adds:**

- A post-marketing pharmacovigilance study of 2074 participants conducted in the EU found that transaminase elevations at ≥3 times the ULN occurred in 3.6% of tolvaptan-treated patients, with the greatest likelihood occurring within the first 18 months of treatment.
- No Hy's law cases indicative of severe liver injury or liver transplantations due to tolvaptan use have been reported to date in this study.
- Tolvaptan was interrupted or withdrawn as recommended per labelling in most participants (91%) with confirmed transaminase elevations ≥3 times the ULN.

**Potential impact:**

- These findings support the importance of regular liver chemistry monitoring per tolvaptan labelling.
- Prompt detection and management of transaminase elevations mitigates the risk of severe liver injury and adverse outcomes.
- Continued post-marketing pharmacovigilance activity is required to better understand tolvaptan liver safety.

## INTRODUCTION

Tolvaptan is the only disease-specific pharmacotherapy approved for autosomal dominant polycystic kidney disease (ADPKD). In the European Union (EU), tolvaptan is indicated to slow the progression of cyst development and renal insufficiency in patients at chronic kidney disease (CKD) stages G1–G4 and with evidence of rapidly advancing disease [1].

The first pivotal clinical trial of tolvaptan, TEMPO 3:4 (NCT00428948), demonstrated therapeutic efficacy on parameters including estimated glomerular filtration rate and total kidney volume [2]. Review of the trial database by an independent data monitoring committee identified an imbalance between the tolvaptan and placebo arms in the proportion of participants who experienced elevations in alanine aminotransferase (ALT) >3 times the upper limit of normal (ULN), i.e. 4.4% for tolvaptan versus 1.0% for placebo [3]. Additionally, three participants in TEMPO 3:4 and its extension study TEMPO 4:4 met criteria for severe drug-induced liver toxicity, known as Hy's law [i.e. serum ALT or aspartate aminotransferase (AST) ≥3 times the ULN and total bilirubin >2 times the ULN, in the absence of cholestasis and without any other reason to explain the elevations], which carries a high risk (≈10%) of acute liver failure or orthotopic liver transplant [4]. Liver chemistry monitoring in the tolvaptan clinical development program, which was performed every 4 months in TEMPO 3:4 and initially every 6 months in TEMPO 4:4, was increased in frequency to every 3 months after unblinding of TEMPO 3:4 and finally to once monthly [5].

In the later REPRISE trial (NCT02160145), while there was an imbalance between tolvaptan and placebo in the percentage of patients with ALT >3 times the ULN (5.6% versus 1.2%), no cases met Hy's law criteria, supporting once-monthly liver enzyme monitoring for the prompt detection and management of liver enzyme elevations [5, 6]. Similarly, no Hy's law cases occurred in the follow-up long-term safety extension trial (NCT02251275) [5, 7]. Data from the clinical development program suggest that the first 18 months of tolvaptan therapy are associated with the greatest likelihood of liver enzyme elevations, with the risk decreasing thereafter [3, 5]. The EU labelling for tolvaptan requires once-monthly monitoring for the first 18 months of treatment and once every 3 months subsequently [1].

Trial data supporting the importance of once-monthly liver enzyme monitoring for the first 18 months of tolvaptan therapy have recently been corroborated by interim post-marketing pharmacovigilance data from the USA, where a Risk Evaluation and Mitigation Strategy (REMS) requires liver chemistry monitoring before the start of treatment, at 2 and 4 weeks after tolvaptan initiation, then monthly during the first 18 months of treatment followed by once every 3 months [8]. To further characterize the liver safety profile of tolvaptan in the post-marketing setting, an interim analysis was performed for the post-authorization safety study (PASS) required as a condition of tolvaptan regulatory approval in the EU.

## MATERIALS AND METHODS

### Study design and eligibility

The PASS study was a prospective, observational study of patients receiving tolvaptan therapy for ADPKD in the real-world clinical context. In the EU countries where tolvaptan is available, certified prescribers who completed the required tolvaptan educational training were invited to participate and enrol patients into the study. Patients were eligible for inclusion if they had been prescribed tolvaptan for ADPKD by the appropriately certified prescriber, were tolvaptan naïve (had never taken tolvaptan, including as part of a clinical trial) and were willing and able to provide informed consent or legal guardian consent, understand the requirements of participation in the study and comply with the study and data collection processes.

The PASS commenced with enrolment of the first patient in October 2016, and the database lock date for this interim analysis was 15 April 2022.

### Endpoints

The primary study endpoint was the incidence of patients with acceptable transaminase levels at screening who experienced transaminase elevations (i.e. ALT or AST ≥3 times the ULN) or an adverse event consistent with hepatotoxicity. A secondary endpoint was the incidence of patients who met Hy's law laboratory criteria (ALT or AST ≥3 times the ULN and total bilirubin >2 times the ULN in the absence of elevated alkaline phosphatase) [4].

### Assessments

Patients were evaluated for eligibility at the initial visit. Study data were obtained through physician records collected as part of the routine standard of care, with the data entered on electronic case report forms (eCRFs).

As per the requirements of the tolvaptan EU Summary of Product Characteristics (SmPC), during the first 18 months of tolvaptan therapy, confirmation of the completion of monthly liver function testing was recorded. After 18 months of treatment, testing for hepatic transaminases and bilirubin should continue at 3-month intervals. Clinical data, including liver function test results, were recorded on the eCRF following each visit. Patients who developed elevated liver enzyme levels after initiating tolvaptan were monitored and managed as outlined in the SmPC, based on the criteria specified there for increased frequency of testing, treatment interruption and possible permanent discontinuation [1]. In addition to laboratory testing, adverse events suggestive of liver injury, such as fatigue, anorexia, nausea, right upper abdominal discomfort, vomiting, fever, rash, pruritus, icterus, dark urine or jaundice, were collected during the study.

### Analyses

The analyses presented here are descriptive summary statistics.

### Ethical conduct

This study was conducted under good pharmacovigilance practices and good pharmacoepidemiology practices established by the International Society for Pharmacoepidemiology, the Declaration of Helsinki and its amendments and national authorities. In addition, the study was performed in accordance with the European Network of Centres for Pharmacoepidemiology and Pharmacovigilance *Guide on Methodological Standards in Pharmacoepidemiology* and the *2012 Guideline on Good Pharmacovigilance Practices: Module VIII—Post-Authorisation Safety Studies* [9, 10]. Each study site obtained approval from an institutional review board or independent ethics committee according to regional requirements.

At the initial (screening) visit, the patients or their legal guardians provided written informed consent. Enrolment was voluntary and patients were free to withdraw consent at any time. The decision to initiate use of tolvaptan was made independently by the patient and healthcare provider and was not mandated by the study design or protocol.

## RESULTS

### Study population

As of the data cut-off date (15 April 2022), 2185 patients were screened for study eligibility and 2118 were enrolled. Accordingly, target study enrolment (2100 participants) was achieved. Of the 2118 enrolled, 2074 (97.9%) participants took at least one dose of tolvaptan and were included in the safety analysis population.

Patients were recruited from Austria, Belgium, Finland, France, Germany, Ireland, Italy, the Netherlands, Norway, Spain, Sweden, Switzerland and the United Kingdom. The overall mean age of participants was 43.0 years [standard deviation (SD) 10.5] with 72.9% <50 years of age and 27.1% ≥50 years of age (Table 1). Nearly equal numbers of male and female participants were enrolled. Most participants were White (92.1%). Baseline CKD stages in the safety analysis population were predominantly G2 (27.2%), G3a (22.6%) and G3b (23.8%), with smaller percentages in G1 (14.8%), G4 (6.1%) and G5 (3 participants; 0.1%). Tolvaptan is indicated for patients in stages G1–G4 at initiation of treatment.

**Table 1:** Participant baseline characteristics (*N* = 2118).

Variable	Values
Sex, *n* (%)	
Female	1028 (48.5)
Male	1081 (51.0)
Sex missing	9 (0.4)
Age (years)	
Mean (SD)	43.0 (10.5)
Range	18–97
Age group (years), *n* (%)	
<50	1543 (72.9)
≥50	573 (27.1)
Age missing	2 (0.1)
White race, *n* (%)	1951 (92.1)
Height (cm)	
Mean (SD)	174.2 (10.3)
Range	115–208
Weight (kg)	
Mean (SD)	82.1 (18.1)
Range	34–175
Total kidney volume (ml)	
Mean (SD)	1933 (1152)
Median	1645
Range	13–8030
CKD stage	2074^a^
G1	307 (14.8)
G2	565 (27.2)
G3a	468 (22.6)
G3b	493 (23.8)
G4	127 (6.1)
G5	3 (0.1)
CKD stage missing	111 (5.4)

a CKD stage information is for participants who took at least one dose of tolvaptan (safety population, *n* = 2074).

### Exposure

As of the data cut-off date, the overall mean duration of tolvaptan exposure in the PASS was 631.3 days (SD 519.2) (approximately 21 months), with a range of 1–1997 days and a median of 527.5 days. Most participants [1922/2074 (92.7%)] initiated therapy at a starting dose of 60 mg (daily split dose of 45 mg + 15 mg), in accordance with the SmPC.

### Incidence of liver injury

**Figure 1:**
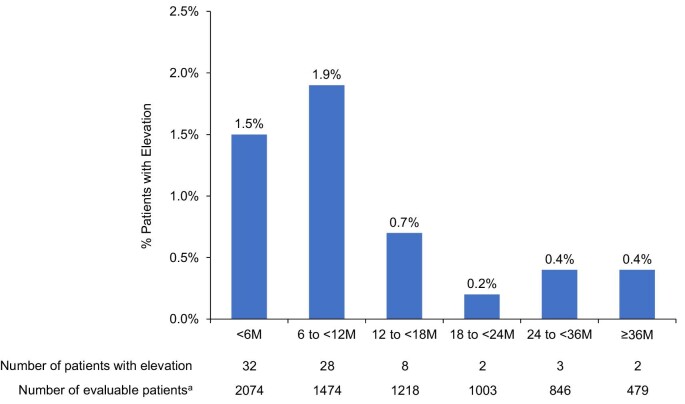
Incidence of ALT or AST ≥3 times the ULN in the safety population (*n* = 2074) by duration of tolvaptan exposure. ^a^The number of patients who were treated with tolvaptan during the time period; the denominator for calculating incidence. M: month.

A total of 223/2074 (10.8%) patients in the safety population experienced ALT or AST ≥3 times the ULN or an adverse event consistent with hepatotoxicity. The group of 223 included 75 (3.6%) with ALT or AST ≥3 times the ULN and 200 (9.6%) who had an adverse event consistent with hepatotoxicity (some participants had both). Fig. 1 shows the incidence of elevated ALT or AST by duration of exposure. Regarding tolvaptan dosage at the time of the initial elevation, 10 participants experienced ALT or AST ≥3 times the ULN at a tolvaptan dose of 60 mg/day, 19 participants at 90 mg/day and 46 participants at 120 mg/day.

**Figure 2:**
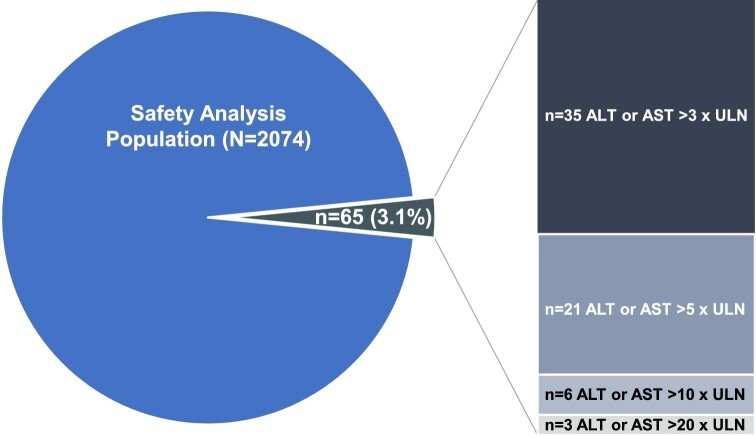
Participants with confirmed ALT or AST ≥3 times the ULN (*n* = 65) by peak level of elevation.

At the time of data cut-off, data review of the 75 participants with ALT or AST ≥3 times the ULN indicated that further confirmation from the investigational sites was needed for 10 participants, given that the reference range reported was incorrect. For the remaining 65 (3.1%) participants with confirmed ALT or AST ≥3 times the ULN, the peak elevation in either ALT or AST is shown in Fig. 2.

Of the 65 participants with confirmed ALT or AST ≥3 times the ULN, 15 experienced only the elevation, while 50 reported accompanying liver-related adverse events. Counting by event instead of participant, there were a total of 84 confirmed ALT or AST ≥3 times the ULN elevations and/or liver-related adverse events, including 15 transaminase elevations ≥3 times the ULN that occurred alone and 69 liver-related adverse events. By investigator assessment, most of the 69 adverse events consistent with hepatotoxicity were mild [41/69 (59.4%)], 18 (26.1%) were of moderate severity and 10 (14.5%) were severe. Of the 69 adverse events suggestive of hepatotoxicity in patients with confirmed ALT or AST ≥3 times the ULN, investigators considered 63 to be related and 6 unrelated to tolvaptan.

### Clinical management and outcomes

The action taken with tolvaptan in response to the 84 confirmed ALT or AST elevations ≥3 times the ULN and/or liver-related adverse events was most often tolvaptan interruption (39 events) or withdrawal (20 events), as shown in Table 2. Counting by individual participant instead of by event, tolvaptan was interrupted or withdrawn as recommended per labelling for 59 of the 65 participants (90.8%) with confirmed ALT or AST ≥3 times the ULN.

**Table 2:** Action taken with tolvaptan and outcome at data cut-off for ALT or AST elevations ≥3 times the ULN and/or liver-related adverse events.

Action	Events, *n* (*N* = 84)	Outcome	Events, *n* (*N* = 84)
Interruption	39	Resolved	66
Withdrawal	20	Resolving	7
Dose unchanged	17	Not resolved	6
Dose reduction	5	Not reported	5
Not reported	2		
Up-titrated	1		

At the time of reporting, 66 of the 84 confirmed ALT or AST elevations ≥3 times the ULN and/or liver-related adverse events had resolved, 7 were resolving, 6 were reported as not resolved and the outcome of 5 was not reported (Table 2). For the six adverse events that were not resolved at the time of reporting, tolvaptan had been interrupted for four, the dose had been reduced for one and tolvaptan was withdrawn for one. Tolvaptan was restarted in 27 participants who had interrupted treatment, of whom 20 continued the restarted tolvaptan for >6 months.

### Assessment of severe liver injury

Forty-nine participants in the study experienced elevations in total bilirubin >2 times the ULN. However, none of these total bilirubin elevations were accompanied by ALT or AST levels ≥3 times the ULN; therefore, there were no cases meeting Hy's law laboratory criteria. No deaths attributable to liver injury occurred.

One participant underwent liver transplant for polycystic liver disease after 278 days of tolvaptan treatment. Her liver function test results were within the normal range throughout the duration of tolvaptan therapy, which was withdrawn at the time of the transplant. The investigator assessed the transplant as unrelated to tolvaptan and due to the patient's underlying condition of polycystic liver disease.

## DISCUSSION

We report liver safety data from a large (>2000 participants) post-marketing pharmacovigilance study of tolvaptan over a median follow-up of ≈1.5 years. Similar to two pivotal clinical trials of tolvaptan in the treatment of ADPKD, in which 4.4–5.6% of participants in the tolvaptan study arms had elevations in ALT >3 times the ULN reported [5], liver enzyme elevations were identified in a small percentage of patients treated with tolvaptan in this real-world clinical setting [75/2074 (3.6%) participants with ALT or AST ≥3 times the ULN]. Based on the liver safety signal detected during clinical development, tolvaptan prescribing information provides criteria for healthcare practitioners to detect liver adverse events based on laboratory testing and clinical symptoms, as well as direction on increased intensity of monitoring, treatment discontinuation and, if warranted, restart of therapy in patients who experience liver toxicity [1, 11].

Our study thus far provides reassurance that treating physicians are performing adequate clinical assessment for signs and/or symptoms of liver injury during tolvaptan therapy and undertaking appropriate action when such cases are detected. Tolvaptan was interrupted or withdrawn as recommended per the label in 59 of 65 (91%) patients with confirmed ALT or AST ≥3 times the ULN. Most [59/69 (86%)] of the liver-related adverse events in this population with elevated transaminases were assessed as mild or moderate in severity. These findings indicate that the safety monitoring permits prompt detection and management of liver enzyme elevations to reduce the risk of more severe hepatotoxicity. Most cases (73/84) of ALT or AST ≥3 times the ULN and/or associated liver-related adverse events were resolved or resolving at the time of data cut-off. Further, no cases meeting Hy's law criteria or of liver transplantation related to tolvaptan treatment were identified in this population with a wide spectrum of kidney function.

Of 27 patients who interrupted tolvaptan therapy and reinitiated treatment, 20 continued with reinitiated tolvaptan for >6 months, supporting the appropriateness of label guidance on the possibility of tolvaptan restart for patients in whom increased laboratory values are not considered related to treatment [1, 11].

An interim analysis of US REMS data found that 4 of 6711 enrolled patients had confirmed serious and potentially fatal liver injury, including 1 who met Hy's law criteria. All recovered after tolvaptan discontinuation and no liver transplants or fatalities due to tolvaptan were reported [8]. Those data and the present study underscore the importance of per label liver safety monitoring in patients receiving tolvaptan for the treatment of ADPKD.

Data from the present study are consistent with previous findings that the window of highest risk for liver toxicity is in the first 18 months of tolvaptan therapy [3, 5, 8]. However, it must be acknowledged that approximately half of the PASS participants did not have follow-up beyond 18 months, which is a study limitation. A greater duration of exposure to tolvaptan will need to accumulate in the PASS and REMS studies before any firm conclusions on the timing of liver adverse events over a long-term duration can be definitively established.

Another limitation of this non-interventional study is that data collection was as per the local standard of care and therefore likely to be less comprehensive than in the controlled setting of a clinical trial. Finally, data collection and verification are ongoing, with the possibility that data on the outcomes of drug-induced liver injury cases will be revised when the study is final.

With these caveats, this interim analysis of data from the PASS corroborates the utility of monthly liver function testing for the first 18 months of treatment and every 3 months thereafter to mitigate the risk of severe liver-related adverse events with tolvaptan. In this study, based on real-world clinical practice, regular safety monitoring facilitated prompt detection of drug-induced liver injury and tolvaptan interruption or withdrawal to reduce the potential for serious and potentially fatal liver injury.

## Data Availability

To submit inquiries related to Otsuka clinical research or to request access to individual participant data (IPD) associated with any Otsuka clinical trial, please visit https://clinical-trials.otsuka.com/. For all approved IPD access requests, Otsuka will share anonymized IPD on a remotely accessible data-sharing platform.
